# Asymptomatic fluid overload predicts survival and cardiovascular event in incident Chinese peritoneal dialysis patients

**DOI:** 10.1371/journal.pone.0202203

**Published:** 2018-08-13

**Authors:** Jack Kit-Chung Ng, Bonnie Ching-Ha Kwan, Kai-Ming Chow, Wing-Fai Pang, Phyllis Mei-Shan Cheng, Chi-Bon Leung, Philip Kam-To Li, Cheuk-Chun Szeto

**Affiliations:** Carol and Richard Yu Peritoneal Dialysis Research Centre, Department of Medicine & Therapeutics, Prince of Wales Hospital, The Chinese University of Hong Kong, Shatin, Hong Kong SAR, China; University of Utah School of Medicine, UNITED STATES

## Abstract

**Background:**

Fluid overload is common among asymptomatic peritoneal dialysis (PD) patients. We aim to determine the prevalence and prognostic significance of fluid overload, as measured by bioimpedance spectroscopy, in asymptomatic incident PD patients.

**Methods:**

We performed a single-center study on 311 incident PD patients. Volume status was represented by the volume of overhydration (OH), OH/extracellular water (ECW) ratio, ECW/total body water (TBW) ratio, and ECW to intracellular water (ICW) ratio (E:I ratio). Patient survival, technique survival and cardiovascular event-free survival were determined.

**Results:**

The median period of follow up was 27.3 months. Fluid overload was present in 272 patients (87.5%) when defined as OH volume over 1.1L. All hydration parameters significantly correlated with Charlson Comorbidity Index, and inversely with total Kt/V, and serum albumin. Multivariate cause-specific Cox analysis showed that volume status independently predicted patient survival; every 0.1 unit increase in E:I ratio was associated with 24.5% increase in all-cause mortality (adjusted cause-specific hazard ratio [ACSHR] 1.245, p = 0.002). Hydration status was also an independent predictor of cardiovascular event-free survival after excluding hospital admission for congestive heart failure; each 0.1 unit increase in E:I ratio was associated with 18.7% decrease in cardiovascular event-free survival (ACSHR 1.187, p = 0.011). In contrast, hydration parameters were not associated with technique survival.

**Conclusions:**

Fluid overload is common in asymptomatic incident PD patients and is a strong predictor of patient survival and cardiovascular event. The impact of bioimpedance spectroscopy-guided fluid management on the outcome of PD patients deserves further study.

## Introduction

Fluid overload is common among patients on peritoneal dialysis (PD), and it represents an important non-traditional risk factor of cardiovascular diseases in this group of patients [1]. Previous studies showed that clinically overt fluid overload is associated with decrease in patient and technique survival [2, 3], excess peritonitis rate (with enteric organisms) [4], increase in left ventricular mass [5] and impaired quality of life [6]. However, assessment of volume status is frequently unreliable by traditional methods. In this regard, bioimpedance spectroscopy provides a non-invasive and reproducible method to estimate hydration status by measuring resistance and reactance of the body under the flow of electrical current [7].

Recently, there is growing evidence that occult fluid overload is present in a substantial number of asymptomatic PD patients [8]. Over one-third of incident PD patients, who are considered euvolemic or dehydrated by clinical assessment, have fluid overload by bioimpedance spectroscopy measurement [9]. However, most of the published studies focus on prevalent PD patients, and the impact of fluid overload on prognosis is usually confounded by congestive heart failure (CHF) [3]. In the present study, we evaluated the prevalence, risk factors, and prognostic significance of fluid overload in clinically asymptomatic incident PD patients.

## Materials and methods

### Patient selection

This is a retrospective analysis of a prospective cohort of incident PD patients in a single dialysis center. This study was approved by the Joint Chinese University Hong Kong-New Territories East Cluster Clinical Research Ethics Committee. All study procedures complied with the Declaration of Helsinki. We studied all clinically euvolemic patients who initiated PD from November 2009 to November 2014. Patients were excluded from the study if they were unable or refused to give consent, or deemed to have fluid overload by clinical judgement.

After informed consent, we performed bioimpedance spectroscopy study around one month after the patient was stable on PD. Dialysis adequacy and nutritional status were determined simultaneously. In addition, background clinical and laboratory data were reviewed. The load of comorbid conditions was determined by the modified Charlson’s Comorbidity Index (CCI) [10].

### Bioimpedance spectroscopy study

We used a validated multi-frequency bioimpedance spectroscopy device (Body Composition Monitor [BCM], Fresenius Medical Care, Germany) as previously reported in healthy subjects and dialysis patients [11, 12]. Briefly, electrodes were attached to one hand and one foot with the patient in a supine position. All measurements were performed with 2 liters of indwelling PD fluid since previous study suggested that the presence of peritoneal dialysate had insignificant effect on BCM measurement [13]. In essence, BCM estimates the electrical resistance by applying alternative current at 50 different frequencies over a range from 5 to 1000kHz. While low-frequency current only passes through extracellular space and gives an estimation of extracellular water (ECM), high-frequency current passes through cell membrane and measures total body water (TBW), which allows extrapolation of intracellular water (ICW) at the same time. Based on a validated physiological model, other body composition parameters, namely, lean tissue mass (LTM), adipose tissue mass (ATM), and volume of overhydration (OH) can be derived from TBW and ECW [14]. We also calculated composite indices including OH/ECW, ECW/TBW and ECW to ICW ratio (E:I ratio).

Although OH volume, OH/ECW, ECW/TBW and E:I ratio were all commonly adopted hydration parameters, there was no consensus on which was a stronger predictor of clinical outcomes. Fluid overload, when defined as ECW/TBW ≥0.40, was associated with significant increase in all-cause mortality and marginal increase in cardiovascular mortality in prevalent PD patients [3]. However, it is argued that water predominates in extracellular space in adipose tissue, which leads to an overestimation of ECW/TCW in obese people irrespective to the hydration status [15]. For similar reason, the proportion of ATM and LTM may limit the accuracy of E:I ratio. On the other hand, OH volume, expressed in liters, provides an estimate of degree of fluid overload and guides the clinician to set the ‘dry weight’. Our previous study also confirmed the clinical importance of OH volume by demonstrating a significant association between arterial stiffness and fluid overload defined as OH ≥1.1L [8]. Nevertheless, the same OH volume may have different implication in patients with different body weight [2]. Normalization of OH with ECW had been proposed and a cut-off of >7% was used in the EuroBCM study [16]. Yet it was worth to note that this cut-off was derived from a group of hemodialysis (HD) patients undergoing body composition measurements [17]. In the present study, we examined the prognostic value of the four aforementioned hydration indices.

### Dialysis adequacy and nutritional assessment

Dialysis adequacy was assessed by 24-hour collection of peritoneal dialysate and urine. Weekly Kt/V was calculated by the standard method. Residual glomerular filtrate rate (GFR) was determined by the mean of 24-hour urinary urea and creatinine clearance. Ultrafiltration volume and residual urine volume were also recorded. Nutritional status was represented by serum albumin level and normalized protein nitrogen appearance (NPNA) as determined by the Bergstrom’s formula [18].

### Outcome measures

All patients were followed until 31^st^ December 2015. Primary outcome measures are patient survival, technique survival, and cardiovascular event-free survival. For patient survival, transfer to hemodialysis, kidney transplantation, transfer to other center, and recovery of renal function were considered as competing events. Death and transfer to long-term HD were considered as events for technique survival, while kidney transplantation, transfer to other center, and recovery of renal function were considered as competing events.

Cardiovascular event was defined as acute coronary syndrome, peripheral vascular disease, stroke, and CHF that required hospital admission. Notably, CHF, which was directly related to volume status, was included as cardiovascular endpoint in previous study [3]. Whether asymptomatic fluid overload remains a predictor of hospitalization, after exclusion of CHF, has not been studied. Therefore, cardiovascular event-free survival was also examined after excluding hospitalization for CHF as event. Secondary outcome measures include the number of hospital admission and duration of hospital stay in the first year.

### Statistical analyses

Statistical analysis was performed by the SPSS for Windows software (version 24.0. IBM Corporation, Armonk, NY). Data are presented as mean ± standard deviation (SD) or median (inter-quartile range [IQR]). Correlation between continuous variables was explored by the Pearson correlation. Hydration status was categorized into 3 tertiles for analysis, with a higher tertile indicating more fluid retention. Poisson log-linear regression models were used to determine the independent predictors of hospitalization. In these models, hydration indices as well as variables with a significance of P <0.1 in univariate analysis were included in the final model.

Kaplan-Meier method was used to present patient survival, technique survival, and cardiovascular event-free survival. Log-rank test was used to compare survival curves. Multivariate cause-specific Cox proportional hazard models by the competing risk method were constructed to determine the independent predictors of survival. In these models, hydration indices as well as variables that reached a significance of P ≤0.1 in univariate analysis were included in the final model. A P value of less than 0.05 in the final model was considered significant. All probabilities were two-tailed.

## Results

During the study period, we recruited 311 consecutive incident PD patients; 258 (83.0%) were on continuous ambulatory peritoneal dialysis (CAPD). All patients received only glucose-based PD solution at the beginning of dialysis. Table 1 summarizes the baseline demographics, nutritional status and dialysis adequacy. Table 2 summarizes the baseline body composition and fluid status.

**Table 1:** Baseline demographics data, nutritional status and dialysis characteristics.

No. of patients	311
Sex (M:F)	172:139
Age (year)	58.8 ± 12.2
Body height (cm)	161.3 ± 8.6
Body weight (kg)	65.2 ± 14.1
Body mass index (kg/m^2^)	24.9 ± 4.3
Blood pressure (mmHg)	
Systolic	136.9 ± 20.5
Diastolic	73.6 ± 13.5
Causes of renal failure, no. of cases (%)	
Diabetic nephropathy	157 (50.5%)
Glomerulonephritis	71 (22.8%)
Hypertensive nephrosclerosis	26 (8.4%)
Polycystic kidney	10 (3.2%)
Obstructive uropathy	12 (3.9%)
Others / unknown	35 (11.2%)
Comorbidities, no. of cases (%)	
Diabetes	185 (59.5%)
Ischemic heart disease	57 (18.3%)
Cerebrovascular disease	62 (19.9%)
Peripheral vascular disease	15 (4.8%)
Charlson’s Comorbidity Index	5.8 ± 2.5
Laboratory parameters	
Hemoglobin (g/dL)	9.5 ± 1.5
Albumin (g/L)	34.8 ± 4.6
Peritoneal transport	
D/P creatinine at 4 hour	0.67 ± 0.14
Low transporter (<0.5)	31 (10.5%)
Low-average transporter (0.5–0.65)	102 (34.6%)
High-average transporter (0.66–0.81)	118 (40.0%)
High transporter (>0.81)	44 (14.9%)
MTAC creatinine (ml/min/1.73m^2^)	10.49 ± 5.61
Dialysis adequacy	
Weekly total Kt/V	2.11 ± 0.58
Ultrafiltration volume (L)	0.5 ± 1.0
Residual GFR (ml/min/1.73m^2^)	3.63 ± 2.56
Residual urine volume (L)	1.0 ± 0.7
NPNA (g/kg/day)	1.15 ± 0.27

Abbreviations: D/P, dialysate-to-plasma concentration ratio; GFR, glomerular filtration rate; MTAC, mass transfer area coefficient; NPNA, normalized protein nitrogen appearance.

**Table 2:** Baseline body composition and fluid status.

No. of patients	311
OH (L)	4.07 ± 3.14
OH/ECW	0.21 ± 0.12
ECW/TBW	0.50 ± 0.04
E:I ratio	1.01 ± 0.18
Lean tissue mass (kg)	38.9 ± 10.9
Adipose tissue mass (kg)	20.2 ± 11.3

Abbreviations: OH, overhydration volume; TBW, total body water; ICW, intracellular water; ECW extracellular water; E:I ratio, the ratio of ECW to ICW.

Among the 311 patients, fluid overload was present in 272 (87.5%) when defined as OH volume >1.1 liters, 274 (88.1%) when defined as OH/ECW ratio >7%, and 305 (98.1%) when defined as ECW/TBW ratio ≥40%. The OH volume had close internal correlations with ECW/TBW (r = 0.76, P <0.0001), and E:I ratio (r = 0.77, P <0.0001). All hydration parameters correlated significantly with CCI, total Kt/V, and albumin level (Table 3). Hydration parameters also had significant but modest correlations with age, body weight, body mass index, systolic blood pressure, transporter status and residual GFR (Table 3). In contrast, hydration status had no significant correlation with ultrafiltration volume. Although EBW/TBW and E:I ratio were weakly associated with residual urine volume, such correlation was not observed with OH and OH/ECW.

**Table 3:** Relation between hydration parameters and demographics, comorbidities, dialysis characteristics.

	OH		OH/ECW		ECW/TBW		E:I ratio	
	r	P-value	r	P-value	r	P-value	r	P-value
Age (year)	0.003	0.96	0.07	0.26	0.30	<0.0001	0.28	<0.0001
BW (kg)	0.35	<0.0001	0.11	0.051	0.16	0.004	0.16	0.004
BMI (kg/m^2^)	0.25	<0.0001	0.07	0.25	0.24	<0.0001	0.23	<0.0001
SBP (mmHg)	0.20	<0.0001	0.26	<0.0001	0.25	<0.0001	0.25	<0.0001
CCI	0.15	0.01	0.18	0.001	0.36	<0.0001	0.34	<0.0001
D/P4_Cr_	0.23	<0.0001	0.25	<0.0001	0.18	0.002	0.18	0.003
Albumin (g/L)	-0.36	<0.0001	-0.43	<0.0001	-0.36	<0.0001	-0.36	<0.0001
Kt/V	-0.26	<0.0001	-0.19	0.001	-0.12	0.04	-0.13	0.03
Residual GFR (ml/min)	-0.07	0.22	-0.14	0.02	-0.15	0.01	-0.17	0.005
UF (L)	0.004	0.95	-0.01	0.89	-0.02	0.69	-0.01	0.94
Residual urine output (L)	-0.004	0.94	-0.09	0.12	-0.14	0.02	-0.15	0.01

Correlation are presented as Pearson correlation coefficient.

Abbreviations: BW, body weight; BMI, body mass index; CCI, Charlson’s Comobidity Index; GFR, glomerular infiltration rate; D/P4_Cr,_ dialysate-creatinine to plasma-creatinine ratio at 4 hour; SBP, systolic blood pressure; UF, ultrafiltration volume; OH, overhydration volume; TBW, total body water; ICW, intracellular water; ECW extracellular water; E:I ratio, the ratio of ECW to ICW.

### Patient and technique survival

**Fig 1:**
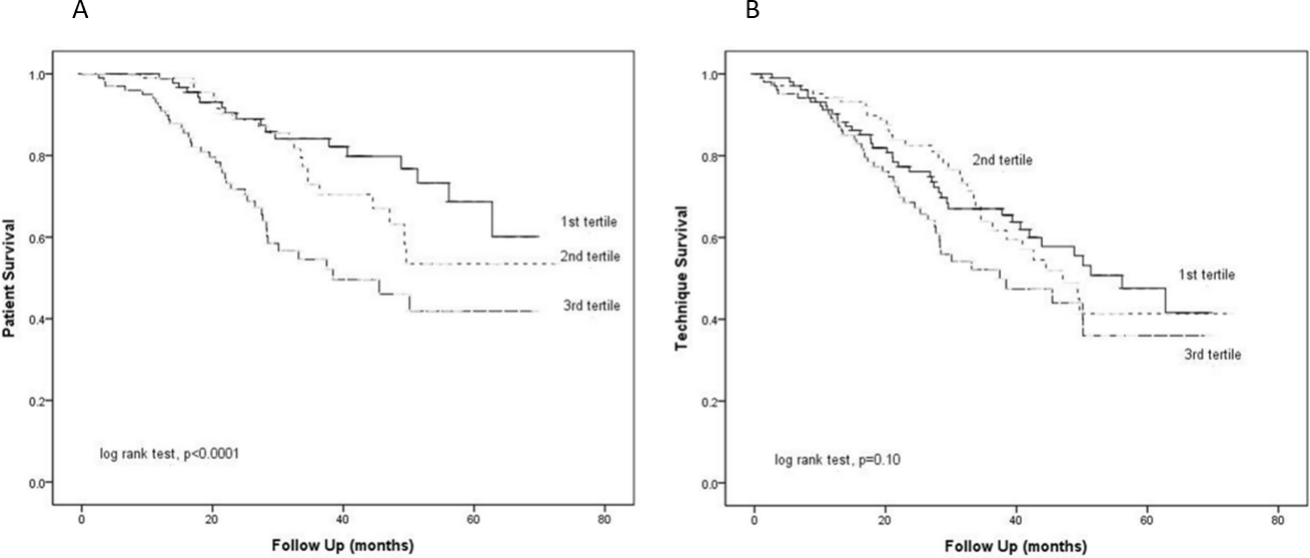
**Kaplan-Meier plot of (A) patient survival; and (B) technique survival.** Patients were divided into tertiles according to their baseline E:I ratio (1^st^ tertile: ≤0.91; 2^nd^ tertile >0.91–1.07; 3^rd^ tertile >1.07). Data were compared by the log rank test.

All patients were followed for a median of 27.3 (IQR 17.2–40.8) months. During this period, 81 patients died. Their causes of death were non-peritonitis infections (25 cases), cardiovascular diseases (23 cases), peritonitis (10 cases), cerebrovascular diseases (8 cases), termination of dialysis (5 cases), and malignancy (3 cases). In addition, 17 patients were transferred to long-term hemodialysis, 23 received kidney transplant, 5 were transferred to another center, and 1 had recovery of renal function. At 36 months, patient survival was 84.1%, 72.9% and 54.6% for E:I ratio first, second, and third tertile, respectively (log-rank test, p <0.0001) (Fig 1A). After adjusting for potential confounding factors, multivariate cause-specific Cox regression analysis showed that E:I ratio and CCI were independent predictors of patient survival, while the results of age just fell short of statistical significance (Table 4). The impact on mortality remained similar when other hydration parameters were used instead of E:I ratio for analysis (S1–S3 Tables). On the other hand, technique survival was 67.0%, 63.8% and 52.2% at 36 months for first, second, and third tertiles of E:I ratio, respectively (p = 0.10) (Fig 1B). Multivariate cause-specific Cox regression analysis confirmed that E:I ratio and CCI were independent predictors of technique survival (Table 4). However, similar association with technique survival was not observed when other hydration parameters were used instead of E:I ratio (S1–S3 Tables). After adjusting for the hydration parameter, neither residual GFR nor urine volume was associated with patient or technique survival.

**Table 4:** Multivariate cause-specific Cox proportional regression models for patient survival, technique survival, and cardiovascular event-free survival.

	Patient survival		Technique survival		CV event-free survival, including CHF		CV event-free survival, excluding CHF	
	ACSHR	P value	ACSHR	P value	ACSHR	P value	ACSHR	P value
E:I ratio (per 0.1)	1.245	p = 0.002	1.136	p = 0.046	1.105	p = 0.098	1.187	p = 0.011
CCI (per unit)	1.169	p < 0.0001	1.184	p = 0.0001	1.205	p < 0.0001	1.252	p < 0.0001
albumin (per g/L)	-	-	-	-	0.926	p < 0.0001	0.945	p = 0.017
SBP (per 10 mmHg)	-	-	-	-	1.106	p = 0.017	-	-
Age (per 10 year)	1.310	p = 0.09	-	-	-	-	-	-

Abbreviations: ACSHR, adjusted cause-specific hazard ratio; CHF, congestive heart failure; CV, cardiovascular; E:I ratio, the ratio of extracellular to intracellular water volume; CCI, Charlson’s Comorbidity Index; SBP, systolic blood pressure.

### Cardiovascular events

**Fig 2:**
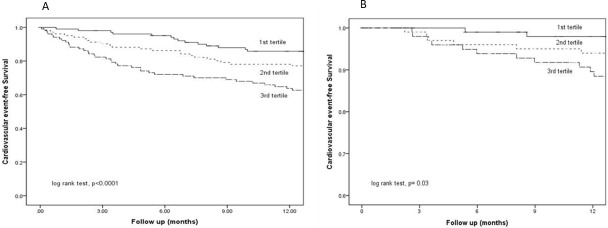
**Kaplan-Meier plot of cardiovascular event-free survival with hospital admission for congestive heart failure (A) included; and (B) excluded**. Patients were divided into tertiles according to their baseline E:I ratio (1^st^ tertile: ≤0.91; 2^nd^ tertile >0.91–1.07; 3^rd^ tertile >1.07). Data were compared by the log rank test.

At 12 months, cardiovascular event-free survival was 85.8%, 78.1% and 63.7% for E:I ratio first, second, and third tertile, respectively (log-rank test, p<0.0001) (Fig 2A). When hospitalization for CHF was excluded, event-free survival was 98.0%, 94.0% and 89.6% at one year for E:I ratio first, second, and third tertile, respectively (log rank test, p = 0.03) (Fig 2B).

By multivariate cause-specific Cox regression analysis, E:I ratio just fell short of statistical significance in predicting cardiovascular event-free survival (Table 4). However, when hospitalization for CHF was excluded, E:I ratio became an independent predictor of event-free survival (adjusted cause-specific hazard ratio 1.187, 95% CI 1.041–1.354, p = 0.011) (Table 4). In this model, every 0.1 unit increment in E:I ratio was associated with 18.7% increase in risk of cardiovascular events. This relationship remained similar when other hydration parameter were used instead of E:I ratio (S1–S3 Tables).

### Relation with hospitalization

During the first year of follow up, there were 515 hospital admissions for a total of 4329 days. This was equivalent to 2.0 admissions and 17.0 days of hospital stay per patient-year. In univariate analysis, the number of hospital admission and the duration of hospital stay in one year were both associated with E:I ratio as well as CCI, SBP and albumin level (Table 5). After adjustment for the aforementioned confounding variables, E:I ratio remained an independent association with the duration of hospital stay in one year, but not the number of hospital admission (Table 5). The relationship remained similar when other hydration parameters were used instead of E:I ratio (data not shown).

**Table 5:** Univariate and multivariate Poisson log-linear regression models for hospitalization in one year.

	Number of hospital admission					Duration of hospital stay				
	univariate		multivariate			univariate		multivariate		
	Exp (B)	P value	Exp (B)	95%CI	P value	Exp (B)	P value	Exp (B)	95%CI	P value
E:I ratio	3.90	p < 0.0001	1.02	0.542–1.922	p = 0.9	12.06	p < 0.0001	2.66	2.148–3.304	p < 0.0001
CCI	1.11	p < 0.0001	1.09	1.042–1.134	p < 0.0001	1.18	p < 0.0001	1.14	1.121–1.156	p < 0.0001
SBP	1.01	p < 0.0001	1.01	1.006–1.015	p < 0.0001	1.01	p < 0.0001	1.00	1.002–1.005	p < 0.0001
albumin	0.91	p < 0.0001	0.92	0.898–0.938	p < 0.0001	0.86	p < 0.0001	0.87	0.867–0.881	p < 0.0001

Abbreviations: Exp(B), exponential of beta value in the model; CI, confidence interval; E:I ratio, the ratio of extracellular to intracellular water volume; CCI, Charlson’s comorbidity index; SBP, systolic blood pressure.

## Discussion

In the present study, fluid overload as detected by bioimpedance spectroscopy was highly prevalent in incident Chinese PD patients despite the absence of clinical features of fluid overload. Moreover, asymptomatic fluid overload was significantly associated with comorbidity load, inadequate dialysis and hypoalbuminemia, but not residual urine volume. We quantified fluid overload by 4 different hydration indices of bioimpedance spectroscopy: OH, OH/ECW, ECW/TBW and E:I ratio, and showed that all of them were independent predictors of mortality in incident PD patients. Irrespective of the hydration parameters used, volume status was an independent predictor of patient survival and cardiovascular event-free survival after hospitalization for CHF was excluded.

Our result is consistent with previous studies, which reported that the prevalence of fluid overload in PD patients ranged from 53.4% to 72.1%, depending on the type of patients studied and the hydration indices adopted [3, 8, 9, 16]. Kang et al conducted a retrospective study of 631 unselected incident PD patients and concluded that a higher edema index (ECW/TBW >0.37) was associated with increase in mortality [19], but cardiovascular event, technique survival and hospitalization were not analyzed. Similarly, a British study on incident as well as prevalent PD patients found that volume status measured by bioimpedance spectroscopy was an independent predictor of overall mortality [2]. Our result provides additional insight to this topic and shows that asymptomatic fluid overload at the initiation of dialysis is an independent predictor of cardiovascular event (after excluding hospital admission for CHF) but not technique survival.

In our present study, asymptomatic fluid overload was independently associated with the length of stay but not the number of hospital admissions (Table 5). However, the practice of hospital admission varies widely in different health care settings, and extrapolation of our result to other patient population may not be appropriate. In our center, renal nurses perform routine home visits shortly after PD training, which may allow early identification of asymptomatic patients who subsequently progressed to overt fluid overload and reduce the number of unplanned hospital admissions. A randomized controlled trial from another Hong Kong center showed that a nurse-led, structured management program significantly improved diet and PD adherence [20], both of which were critical for maintaining optimal fluid status.

In this study, residual GFR and urine volume, which are usually strong predictor of clinical outcome in PD patients, did not predict patient survival after adjusting for volume status. Similarly, O’Lone et al [2] postulated that the lack of association was the result of type II statistical error. We believe this observation represents the phenomenon of model overfitting because the link between residual renal function and clinical outcome is related to the risk of fluid overload, and once body fluid status is adjusted for, the prognostic value of residual renal function would be lost. A recent study further suggested that fluid overload *per se* was independently associated with the rate of residual renal function decline [21]. Taken together, our study suggests that maintaining normal volume status is at least as important as preservation of residual renal function in terms of patient survival.

We performed separate analysis to determine the predictive value of hydration parameters with and without counting hospitalization for CHF as event. Since the differentiation between genuine myocardial dysfunction and volume overload is often difficult, study that include CHF as cardiovascular endpoints may be biased [3]. Our original hypothesis was that the association between fluid overload and cardiovascular events would be attenuated after CHF was excluded. Contrary to what we predicted, volume status was associated with cardiovascular event-free survival only after hospitalization for CHF was excluded. Although the reason behind our findings was not completely understood, published evidence does support the relation between fluid overload and cardiovascular disease. For example, our previous study found that volume of fluid overload correlated with carotid-femoral pulse wave velocity [8]. Another study showed that patients with fluid overload had higher left atrial diameter and carotid artery intima-medial thickness [22]. Our present study is the first to report the relation between asymptomatic fluid overload and cardiovascular events in PD patients.

Our study has several limitations. First, the inherent weakness of a retrospective study makes it impossible to establish the causal relationship between fluid overload and clinical outcome. Second, as a single-center study, our result needs further validation in other patient populations. Third, only baseline BCM measurement was performed. It remains unclear whether longitudinal monitoring of hydration status would have a better prognostic value. In addition, BCM measurement could only be performed around 4 weeks after PD due to logistic reasons. Therefore, the results may not completely reflect the baseline hydration status of the patients. Furthermore, we do not have information regarding the dietary salt and water intake. Liberal oral intake potentially explains the high prevalence of fluid overload in patients with substantial urine volume but may affect clinical outcome independently.

In summary, fluid overload is common in asymptomatic incident PD patients and is a strong predictor of mortality and cardiovascular events. BCM may therefore serve as a simple and non-invasive method to detect subclinical OH at early stage. The impact of bioimpedance spectroscopy-guided fluid management on the outcome of PD patients deserves further study.

## Supporting information

S1 Table Multivariate cause-specific Cox proportional regression models for patient survival, technique survival, and cardiovascular event-free survival (hydration parameter: OH). (DOCX)

S2 Table Multivariate cause-specific Cox proportional regression models for patient survival, technique survival, and cardiovascular event-free survival (hydration parameter: OH/ECW). (DOCX)

S3 Table Multivariate cause-specific Cox proportional regression models for patient survival, technique survival, and cardiovascular event-free survival (hydration parameter: ECW/TBW). (DOCX)
