# Supplementary material for: Asymptomatic fluid overload predicts survival and cardiovascular event in incident Chinese peritoneal dialysis patients
Source: PLoS One. 2018 Aug 13;13(8):e0202203. doi: 10.1371/journal.pone.0202203 (PMC6089451; doi:10.1371/journal.pone.0202203)
Supplement: S1 Table — (DOCX) [file pone.0202203.s001.docx]

S1 table. Multivariate cause-specific Cox proportional regression models for patient survival, technique survival and cardiovascular event-free survival (hydration parameter: OH).

|  | Patient survival | | Technique survival | | CV event-free survival, including CHF | | CV event-free survival, excluding CHF | |
| --- | --- | --- | --- | --- | --- | --- | --- | --- |
|  | ACSHR | P value | ACSHR | P value | ACSHR | P value | ACSHR | P value |
| OH (per L) | 1.096 | p = 0.009 | - | - | 1.057 | p = 0.048 | 1.071 | p = 0.032 |
| CCI (per unit) | 1.138 | p = 0.033 | 1.191 | p < 0.0001 | 1.190 | p < 0.0001 | 1.269 | p < 0.0001 |
| albumin (per g/L) | 0.952 | p = 0.051 | 0.958 | p = 0.055 | 0.940 | p = 0.002 | 0.943 | p = 0.011 |
| SBP (per 10 mmHg) | - | - | - | - | 1.095 | p = 0.033 | - | - |
| Age (per 10 year) | 1.446 | p = 0.015 | - | - | - | - | - | - |

Abbreviations: ACSHR, adjusted cause-specific hazard ratio; CHF, congestive heart failure; CV, cardiovascular; OH, volume of overhydration; CCI, Charlson’s Comorbidity Index; SBP, systolic blood pressure.
